# Drag reduction mechanism of *Paramisgurnus dabryanus loach* with self-lubricating and flexible micro-morphology

**DOI:** 10.1038/s41598-020-69801-6

**Published:** 2020-07-30

**Authors:** Liyan Wu, Huan Wang, Yuqiu Song, Benhua Zhang, Yan Xu, Cuihong Liu, Yuying Yan

**Affiliations:** 1 College of Engineering, Shenyang Agricultural University, Shenyang, 110866 People’s Republic of China; 2 Faculty of Engineering, University of Nottingham, Nottingham, NG7 2RD UK; 3 Research Centre for Fluids and Thermal Engineering, University of Nottingham Ningbo China, Ningbo, 315100 People’s Republic of China

**Keywords:** Mechanical engineering, Soft materials, Structural materials, Fluid dynamics

## Abstract

Underwater machinery withstands great resistance in the water, which can result in consumption of a large amount of power. Inspired by the character that loach could move quickly in mud, the drag reduction mechanism of *Paramisgurnus dabryanus loach* is discussed in this paper. Subjected to the compression and scraping of water and sediments, a loach could not only secrete a lubricating mucus film, but also importantly, retain its mucus well from losing rapidly through its surface micro structure. In addition, it has been found that flexible deformations can maximize the drag reduction rate. This self-adaptation characteristic can keep the drag reduction rate always at high level in wider range of speeds. Therefore, even though the part of surface of underwater machinery cannot secrete mucus, it should be designed by imitating the bionic micro-morphology to absorb and store fluid, and eventually form a self-lubrication film to reduce the resistance. In the present study, the *Paramisgurnus dabryanus loach* is taken as the bionic prototype to learn how to avoid or slow down the mucus loss through its body surface. This combination of the flexible and micro morphology method provides a potential reference for drag reduction of underwater machinery.

## Introduction

Saving energy and reducing its consumption have long been an important topic that had drawn much attention of researchers. Energy consumption is also a significant factor in the application of underwater machinery such as ships and underwater vehicles, as well as civilian fishing boats, equipment for paddy fields, etc. The underwater machinery had a long-term contact with water. Thus, it had to overcome the travel resistance caused by fluid pressure, surface friction, etc. Therefore, the research on drag reduction has great significance for enhancing the dynamic performance of underwater machinery and reducing energy consumption.

Conventional drag reduction methods for fluid interfacial layer included adopting polymer additives, micro-structured surfaces, surface coating, micro-bubble layer, flexible wall, etc.^1–6^. Bionic groove drag reduction technology has been well recognized as drag reduction method. Walsh et al. proposed that v-shaped grooves had better drag reduction performance and the maximum drag reduction rate reached 8% under turbulent conditions^7,8^. Bechert et al. compared and analyzed the grooves of triangular, semicircular, blade and three-dimensional, and found that the viscous resistance of pipelines with v-shaped grooves was 9.9% lower than that with smooth surface. A thin film with v-grooves was coated 70% on the plane's surface to reduce drag during flight and saved 1–2% on fuel^9,10^. Dolphin was one of the reported creatures with a flexible adaptive drag-reduction function, and it could break the theoretical swimming speed limit due to a rough body surface^11^. The study on bionic drag reduction of shark mainly focused on the microscopic groove structure on its scales surface. Amy et al. studied the short-fin Mako shark and found that the scales with groove structure could rotate freely within 50° range in order to adapt to different directions of flow fields. In the initial stage of the occurrence of vortex, the variation of scale’s angle could prevent the vortex from further development and finally suppress the occurrence of vortex^12–14^. Inspired by shapes of barchan dunes in deserts, Song et al. found trough numerical simulation that the maximum drag reduction rate of bionic non-smooth surface was 33.63%^15^. Kumagai et al. studied the drag reduction effect of micro-bubbles by injecting airflow into the machinery’s surface to form a layer of micro-bubbles. The method was mostly used on the surface of ships, which could replace the “metal-water” interface with “gas–water” interface, and consequently saved 5–15% of energy of the military vessels^16^. Researchers also found that the surface micro structures of bird feathers have functions of drag reduction. Chen et al. adopted the replica molding method to form a stripe structure like the bird feather on a polymer material. They found that, when the flow rate was 5.5 m/s, the underwater drag reduction rate of the striped material reached 16%^17^. Inspired by the non-smooth surface of dung beetle and other species, Ren et al. created bionic micro structures on the earth-moving parts of bulldozers^18^. They achieved good drag reduction effect and proposed the drag reduction theory of bionic non-smooth surface and bionic coupling. In bionic study on the drag reduction of fish scale, Wu et al. designed a fish scale-shaped floating plate of the transplanter. A flow field with vortexes was formed in the interface layer via the scale-like structure on the material’s surface. As a result, a drag reduction rate of 3.014% was obtained^19,20^.

Above studies on the drag reduction of L-shaped, V-shaped, U-shaped and space-V-shaped grooves were deemed on rigid surface. Meanwhile, the bionic grooves only showed drag reduction performance within certain speed range. If the travel speed changed, the drag reduction effect may be simultaneously changed or even lost since the lack of deformation of rigid materials. In addition, numerous creatures in nature have evolved to possess various skills of moving faster. These unique skills provide bionics inspiration for researchers on drag reduction. Namely, drag reduction designs were fulfilled through bionic surface of microscopic morphology, structure and materials. Except for above mentioned methods, some aquatic species also benefit from the secretion of mucus, which provided a good lubrication effect. For instance, loach, eel and squid, etc. could swim freely in mud since their body surface covered by mucus which greatly reduced the resistance. Given this, researchers had collected the mucus secreted by the organisms and painted on the machinery surface, which resulted in a significant drag reduction effect. And the drag reduction results exhibited relation to the non-smooth morphology of the underwater vehicle surface. However, these coatings had poor mechanical strength and the drag reduction function was neither sustainable nor recoverable from failure^21–23^.

Therefore, the ability of forming and maintaining self-lubricating was a meaningful research in the case where the material itself could not secrete mucus. Loaches and other soft body mud creatures could absorb their mucus coating when they were squeezed and scratched by sediments, indicating that the self-adhesion of mucus was excellent. However, as mentioned in the previous literature, the artificially collected and painted mucus gradually slipped off from the materials surface, which demonstrating that secretion of mucus was not enough, and that it was also important to retain mucus for longer time by avoiding or slowing down its loss. Revealing the adsorption and retention mechanisms of mucus was beneficial for application in underwater machinery. This would allow a fluid lubricating film to be continuously formed on the surface of component via adsorbing and storing water. In this paper, the loach was selected as the bionic prototype. Scanning electron microscopy (SEM) and 3D microscope were used to analyze the architectural feature and distribution of the surface micro morphology and the geometrical parameters of the micro-units. Then, *Solidworks* software was used to build a 3D visualization model of micro surface morphology. The Fluent software was used to explore the water adsorption and retention mechanism of the loach’s surface. Numerical simulation of bionic structures with different flexible deformations was carried out, and the variation rule of drag reduction rate and flexibility was explored.

## Methods

### Analysis of bionic prototype

**Figure 1:**
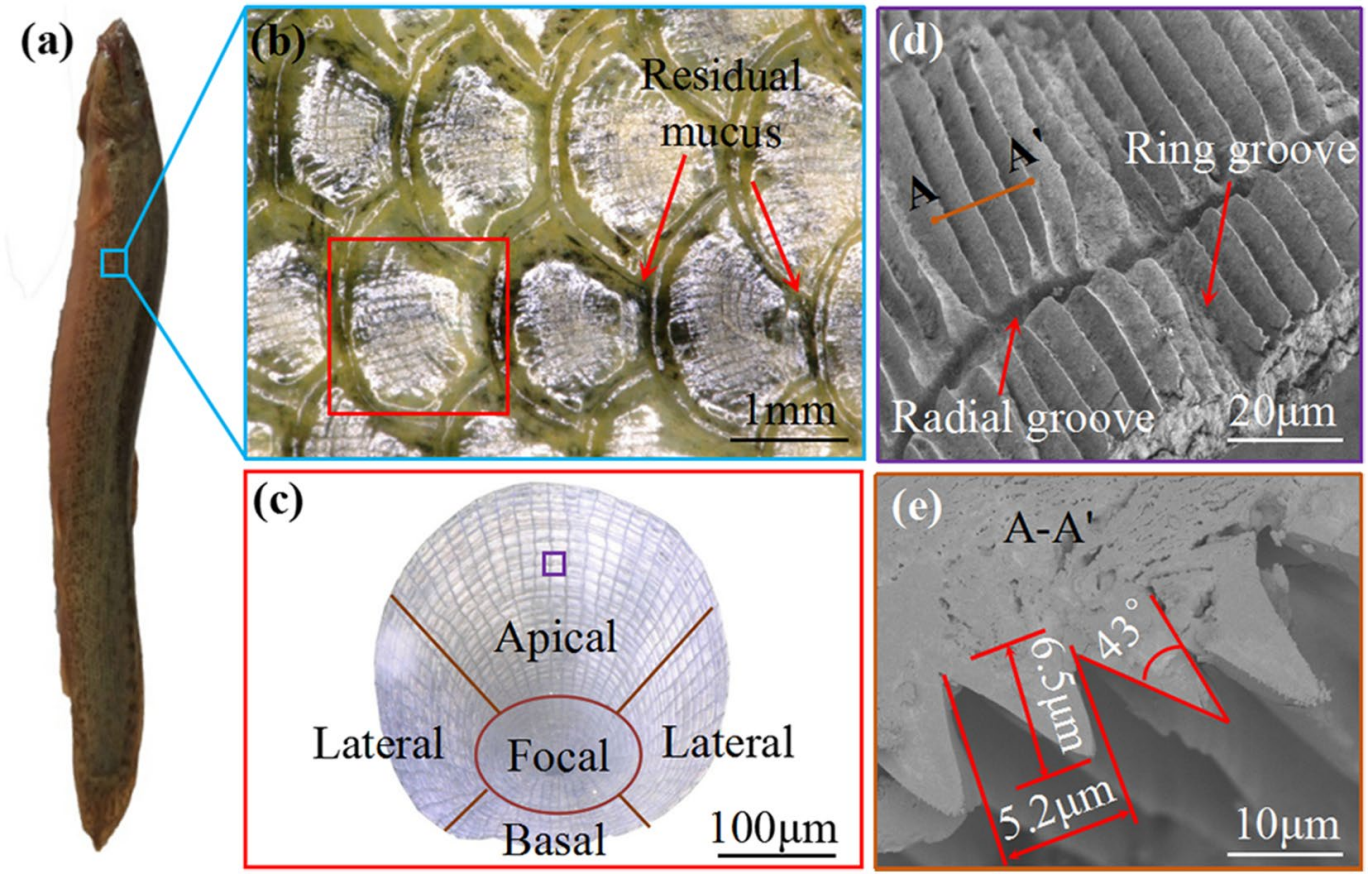
Microscopic morphology of loach body. (**a**) Loach; (**b**) "Scale" morphology; (**c**) Different regions on a single scale; (**d**) Groove structure; (**e**) Cross-section image of grooves.

The pre-treatments for biological prototype include the following: 50 mL of sodium bicarbonate solution with a concentration of 15–20% was first prepared. *Paramisgurnus dabryanus loach* was narcotized with ether and killed without any kinds of cruel autopsy or abuse. Then, the loach was placed in the solution for 10 min to remove the mucus on its body surface. After being taken from the solution, the loach was rinsed with distilled water for 30 min to remove the sodium carbonate crystals remaining on its body. The scales were taken and placed in ethanol with different concentrations (30%, 50%, 70%, 80% and 90%) for 5 min. Afterwards, the scales were cleaned to remove surface impurities with an ultrasonic cleaner. The treated samples were observed by a 3D microscope (VHX-5000) and a SEM (Regulus 8100). The results are shown in Fig. 1. All above treatments definitely complied with the Chinese law on the Protection of Animals. Ethical approval was given by the animal Experimental Ethical Inspection, Shenyang Agricultural University.

Under low magnification, the overall distribution of the surface “scales” can be seen clearly. Each scale is covered with V-shape grooves. The single scale surface can be divided into four regions: apical, focal, basal and lateral. Among these, the focal and basal regions embedded in the skin tissue. The lateral regions of adjacent scales are overlapping arranged (Fig. 1b), and each single scale has shield-like overall shape (Fig. 1c). The apical region is exposed to the body surface and contact with the external fluid media. Therefore this region was selected to be observed by scanning electron microscope (Fig. 1d). There are radiant and circular grooves on apical regions. These grooves could make the scales show great flexibility and elasticity (Fig. 1d). The height of the v-shaped groove is 6.5 μm, the space between adjacent units are 5.2 μm, and the vertex angle is 43° (Fig. 1e).

### Modelling

**Figure 2:**
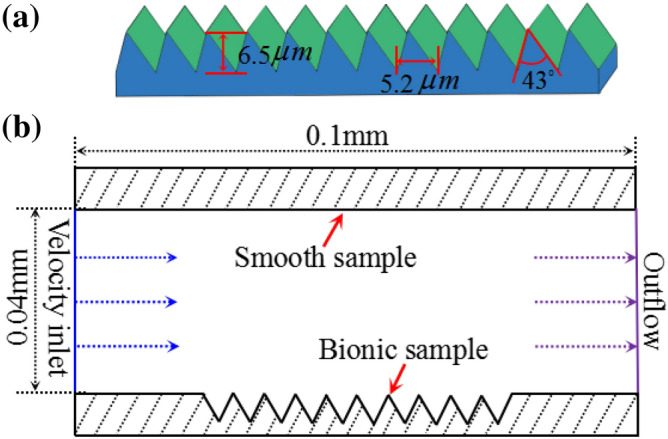
3D model of bionic scales. (**a**) Structure and dimensions of grooves of bionic scales; (**b**) establishment of ICEM bionic Model.

Based on the micro structure analysis of the loach scale, the geometrical structural parameters were measured and a 3D visualization model was built as shown in Fig. 2a. Figure 2a shows the triangle-like grooves and the characteristic parameters were measured: the height of the groove is 6.5 μm; the space between adjacent units were 5.2 μm, and the vertex angle was 43°. In order to analyse the mechanism of fluid absorption and drag reduction, a 2D model with 0.1 mm long and 0.04 mm wide is established through software of Integrated Computer Engineering and Manufacturing Code for Computational Fluid Dynamics (ICEMCFD). The upper and lower boundaries are set as bionic morphology and smooth surfaces, respectively. The left and right sides are set as flow velocity inlet and outflow (Fig. 2b).

## Results and discussion

### Numerical simulation

In this paper, when analyzing the loss prevention or sustainable lubrication of loach surface fluid, water is used instead of mucus, only the maintain and continuously ability of water medium was analyzed. Moreover, if the surface is well maintained to prevent loss of water medium, it must also have a maintenance effect on viscous medium. In the present numerical analysis. Firstly, ICEM software was used to divide the grid, and then it was imported into Fluent. In task page, the Time is set as transient, the Gravitational Acceleration in the Y direction is minus 9.81 m/s^2^. Substitute the value into formula (1), and the result is 5 × 10^3^ < Re < 4 × 10^5^. Select the model $k - \varepsilon$ the fluid medium in the model is liquid–water. Liquid–water is internal fluid with density of 1.0 × 10^3^ kg∙m^−3^ and a dynamic viscosity coefficient of 1.0 × 10^−3^ Pa s. The smooth and bionic surfaces were set to stationary wall surfaces (set as Aluminum). The velocity-inlet is set as 0.05–4 m/s, and then in the Run Calculation panel, the Time Stepping Method is set as Fixed mode, the Time Step Size is 0.001 s, the Number of Time Steps is set as 2,000, and the Max Time Step is set as 50. The calculated result of force is shown in Table 1. Then, the near-wall turbulent intensity and turbulence kinetic energy can be calculated according to Eqs. (1) and (2):

1 $$I = 0.16({\text{Re}} )^{ - 1/8} \left( {{\text{Re}} = {\raise0.7ex\hbox{${\rho uL}$} \!\mathord{\left/ {\vphantom {{\rho uL} \mu }}\right.\kern-\nulldelimiterspace} \!\lower0.7ex\hbox{$\mu $}}} \right)$$

2 $$k = \frac{3}{2}(uI)^{2}$$

**Table 1:** Contrast between the drag forces of the smooth and bionic non-smooth surfaces.

Velocity (ms^−1^)	Smooth surface			Bionic non-smooth surface		
	Pressure drag (N)	Viscous drag (N)	Total drag (N)	Pressure drag (N)	Viscous drag (N)	Total drag (N)
0.05	0	0.0010067	0.0010067	0.0004377	0.0005493	0.0009870
0.5	0	0.0108908	0.0108908	0.004592	0.0060618	0.0106547
1.0	0	0.0237039	0.0237039	0.0099905	0.0132042	0.0231947
1.5	0	0.0383821	0.0383821	0.0163724	0.0212631	0.0376355
2.0	0	0.0547412	0.0547412	0.0237191	0.0301941	0.0539132
2.5	0	0.0726247	0.0726247	0.0320074	0.0398022	0.0718096
3.0	0	0.0919406	0.0919406	0.0412286	0.0500343	0.0912629
3.5	0	0.1126005	0.1126005	0.0513640	0.060840	0.1122040
4.0	0	0.1345099	0.1345099	0.0623983	0.0721853	0.1345836

where, $I$ represents the turbulence intensity; $R{\text{e}}$ the Reynolds number,$\rho$ the fluid density; $u$ the mean velocity of fluid; $k$ the turbulence kinetic energy and $\mu$ the viscosity coefficient; *L* the Characteristic length.

The pressure drag, viscous drag and total drag on smooth surface and bionic surface under different water velocity are shown in Table 1, respectively. Then, the drag reduction rate was calculated according to Eq. (3)

3 $$\eta = \left( {\frac{{F_{ts} - F_{tb} }}{{F_{ts} }}} \right) \times 100\% ,$$

where, $\eta$ represents the drag reduction rate, *F*_tb_ the total drag of bionic surface, *F*_ts_ the total drag of smooth surface.

**Figure 3:**
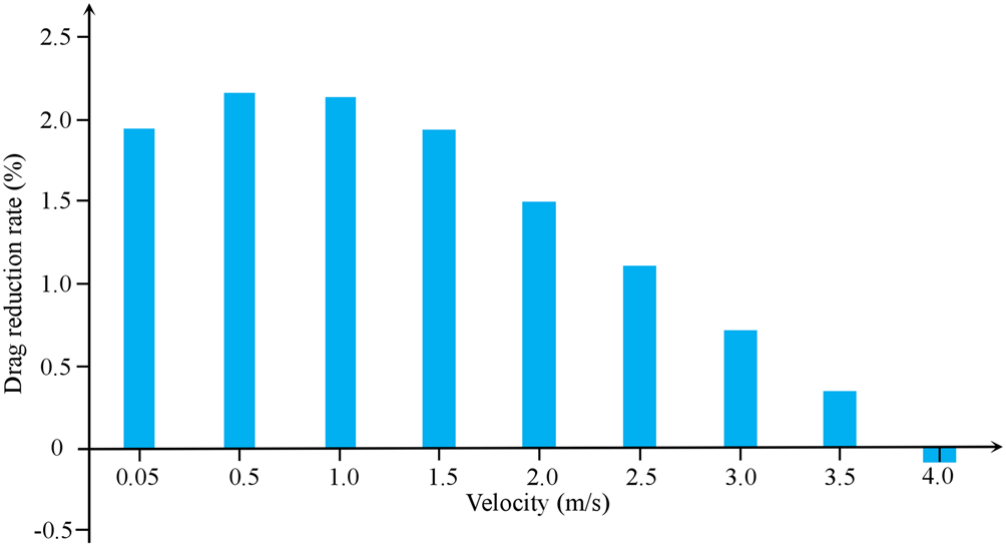
Drag reduction rate at different flow velocity.

**Figure 4:**
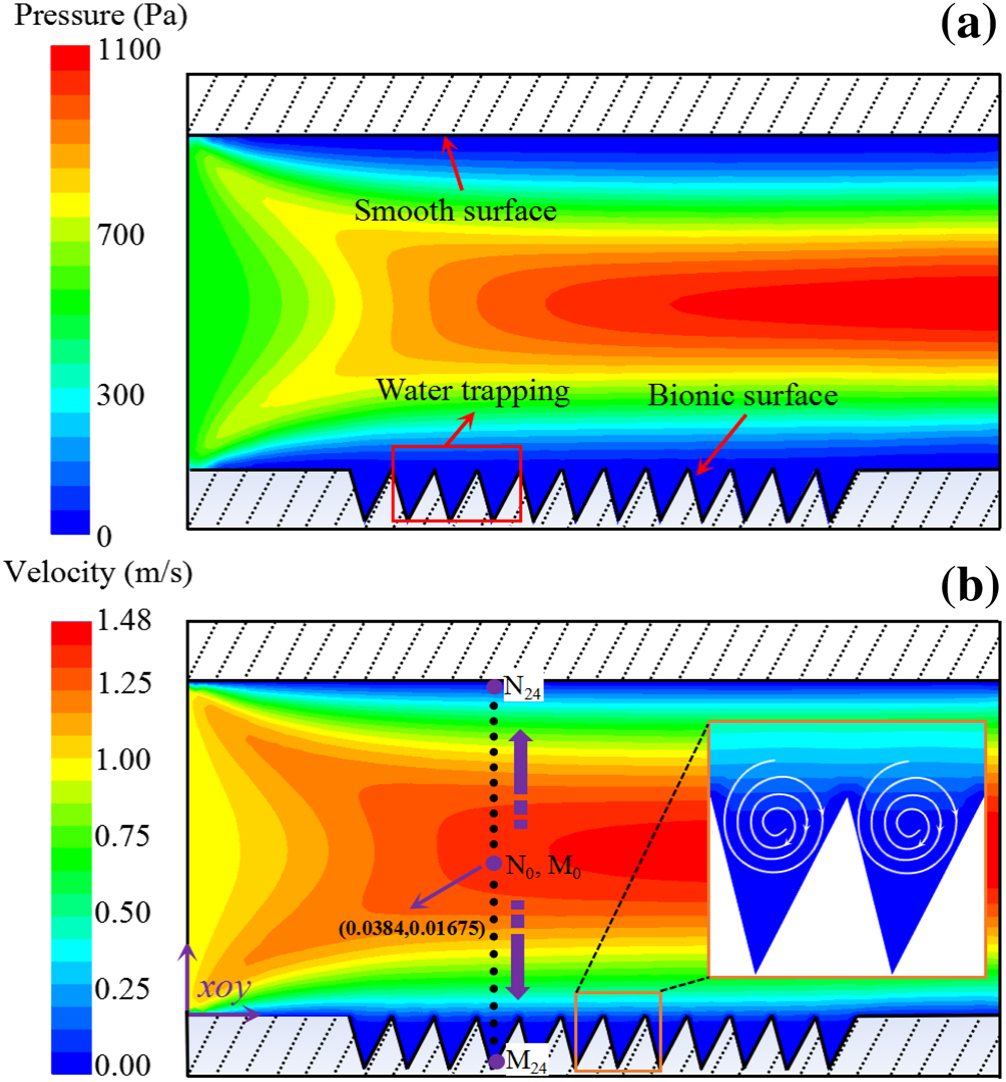
Simulated results of Fluent numerical analysis. (**a**) Distribution of pressure; (**b**) distribution of velocity.

As shown in Fig. 3, the maximum of drag reduction rate appeared at velocity range of 0.5–1.0 m/s, and the drag reduction rate decreases with the increase of velocity. When the speed exceeds 1 m/s, the drag reduction rate decreases sharply. Figure 4 shows the distributions of velocity and pressure in the near-wall region of at flow velocity of 1 m/s.

There are stable low-pressure regions near each micro units, which absorb fluid from surrounding and finally formed a stable “water trapping” region (Fig. 4a). Namely, in these micro groove areas, due to the low pressure, the grooves will absorb water. At the same time, the higher pressure of the surrounding environment will drive the water to flow into these areas. So even if there is a loss of water in the groove, the surrounding water will be replenished constantly. To ensure that there is always some moisture in these groove areas. This creates a phenomenon that seems to trap the water. The “water trapping” units were formed between two adjacent grooves and vortexes with low velocity were formed around the surface of micro structure, exhibiting a clockwise motion as shown in Fig. 4b. The flow above vortex has the same direction with incoming flow, and the flow below vortex eddied to the opposite direction. All adjacent vortex were noninterference. Therefore, the existence of grooves generated the effect of rolling bearings between fluid and the wall surfaces, and the sliding friction was transformed to a rolling friction^24^. But as the speed increases, part of the vortex water was lost. The Rolling bearings effect would be weakened or even destroyed. That is why the drag reduction rate decreased after 1 m s^−1^ in Fig. 3. Under the action of such vortexes, a large amount of fluid with low velocity can accumulate between the adjacent cells and form a layer of water film. Under the joint action of groove micro structure and flow field, the fluid could be continuously absorbed on the surface of micro structure.

In order to analyse the variation of flow field on different surfaces, a series of hypothetical points (M_i_, N_i_) were selected to analyse the fluid velocity, turbulence kinetic energy and turbulence intensity. Each point position of Mi and Ni are symmetric along the axis of the center, as shown in Fig. 4b.

**Figure 5:**
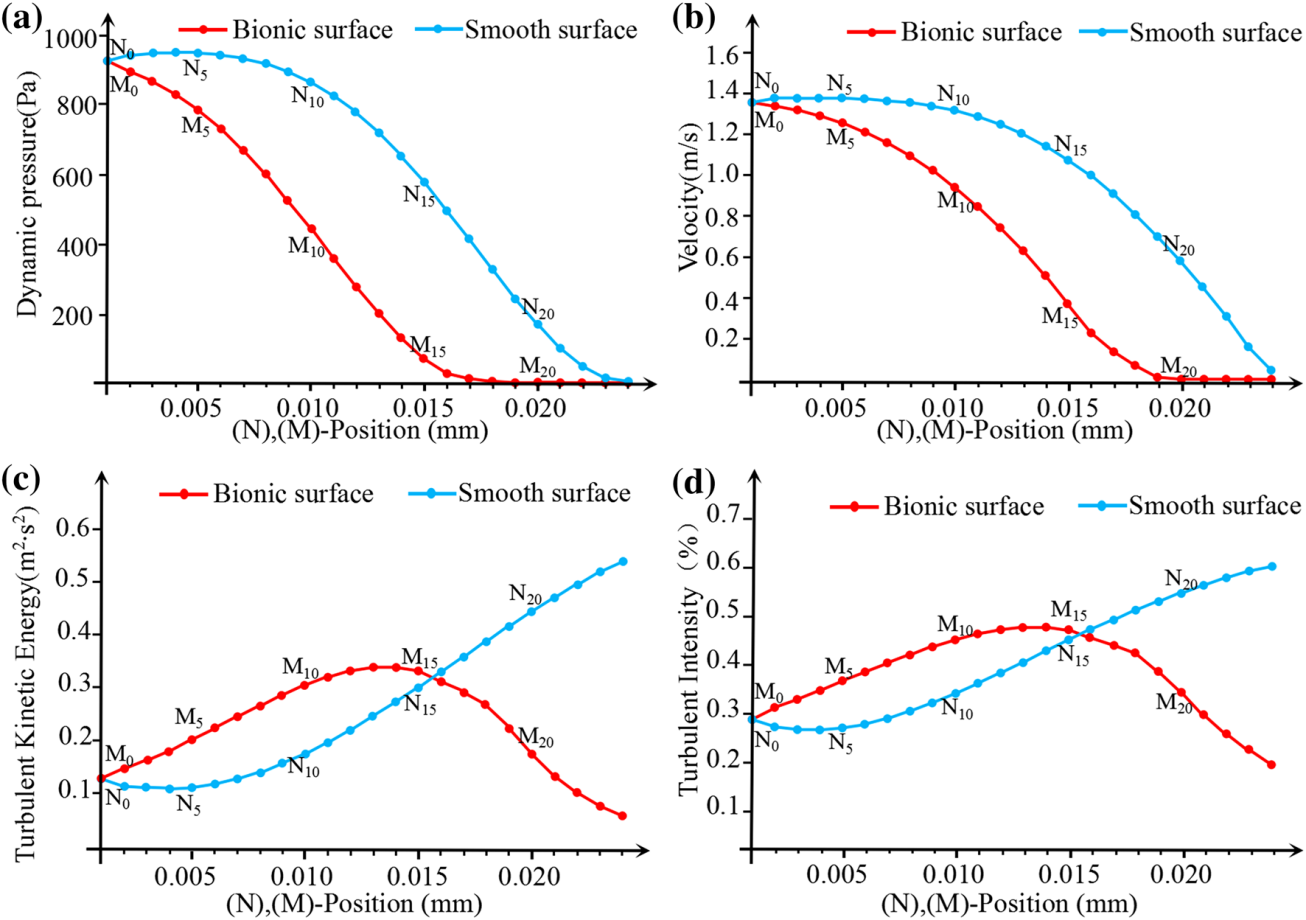
Numerical analysis results. (**a**) Dynamic pressure; (**b**) velocity; (**c**) turbulence kinetic energy; (**d**) turbulence intensity.

Figure 5 shows the variations of the dynamic pressure, velocity, turbulence kinetic energy and turbulence intensity on two types of surfaces. It can be seen that the dynamic pressure and velocities at all points are higher than those equivalents of the smooth surface (5a, 5b). As for the thickness, water membrane on bionic surface is thicker than that on smooth surface. It also can be seen that the velocity gradient of bionic surface was lower than that of smooth surface. So it is easy to conclude that the viscous stress of bionic surface is much lower than the smooth surface. Figure 5c and d show the variation values of turbulence kinetic energy and intensity at every points along the *y* axle. From these results, the turbulence intensity and kinetic energy of bionic surface were both lower than those of smooth surface, and the Reynolds stress of bionic surface was consequent lower than smooth one. The viscous and Reynolds stress of bionic surface was both lower than smooth surface, so the viscous drag of bionic surface was lower than the smooth surface. As part of total drag, viscous drag is reduced by the bionic surface. As another part of the total drag, pressure drag is generated on the bionic non-smooth surface. But the ratio of pressure drag is relative smaller, and the increment of pressure drag less than that decrement of viscous drag (Table 1). Therefore, the total drag is reduced on the bionic surface.

### Analysis of flexible surfaces

**Figure 6:**
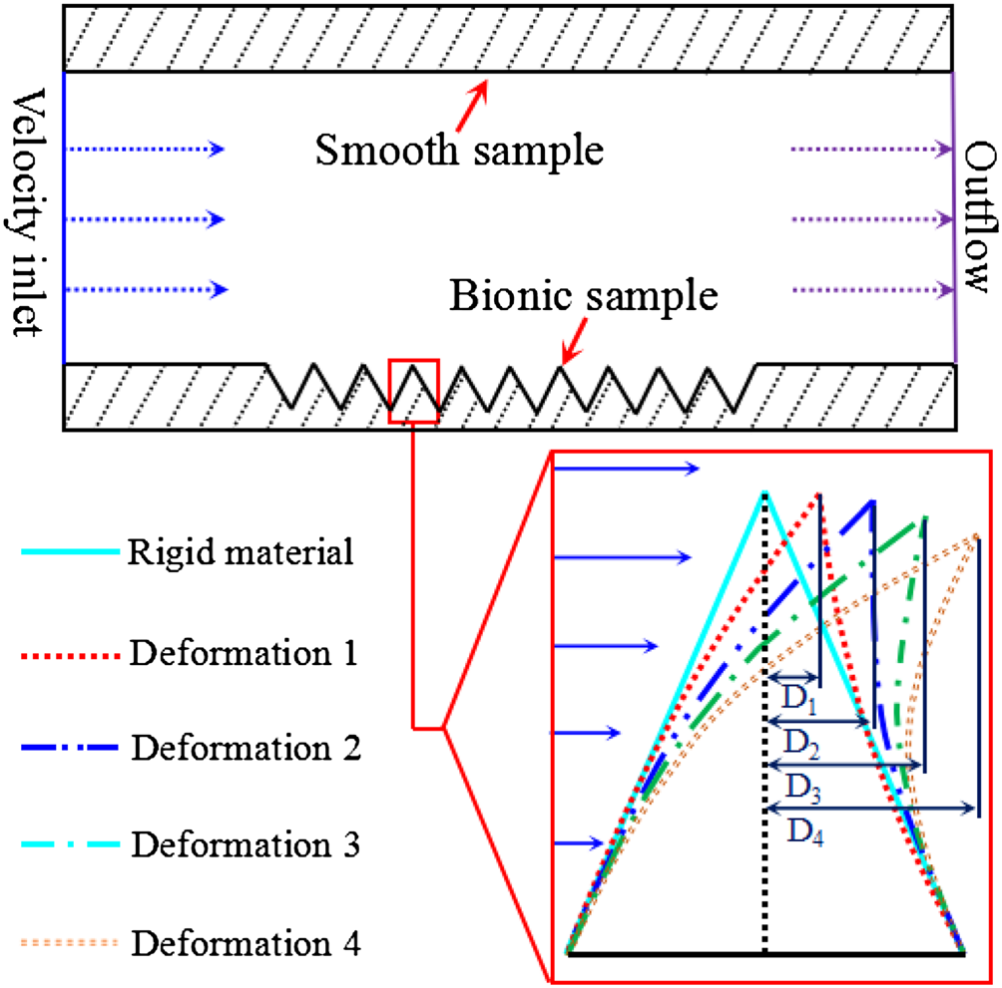
Bionic grooves with different flexible deformations. (D_1_ = 0.7 μm, D_2_ = 1.4 μm, D_3_ = 2.1 μm, D_4_ = 2.8 μm).

**Figure 7:**
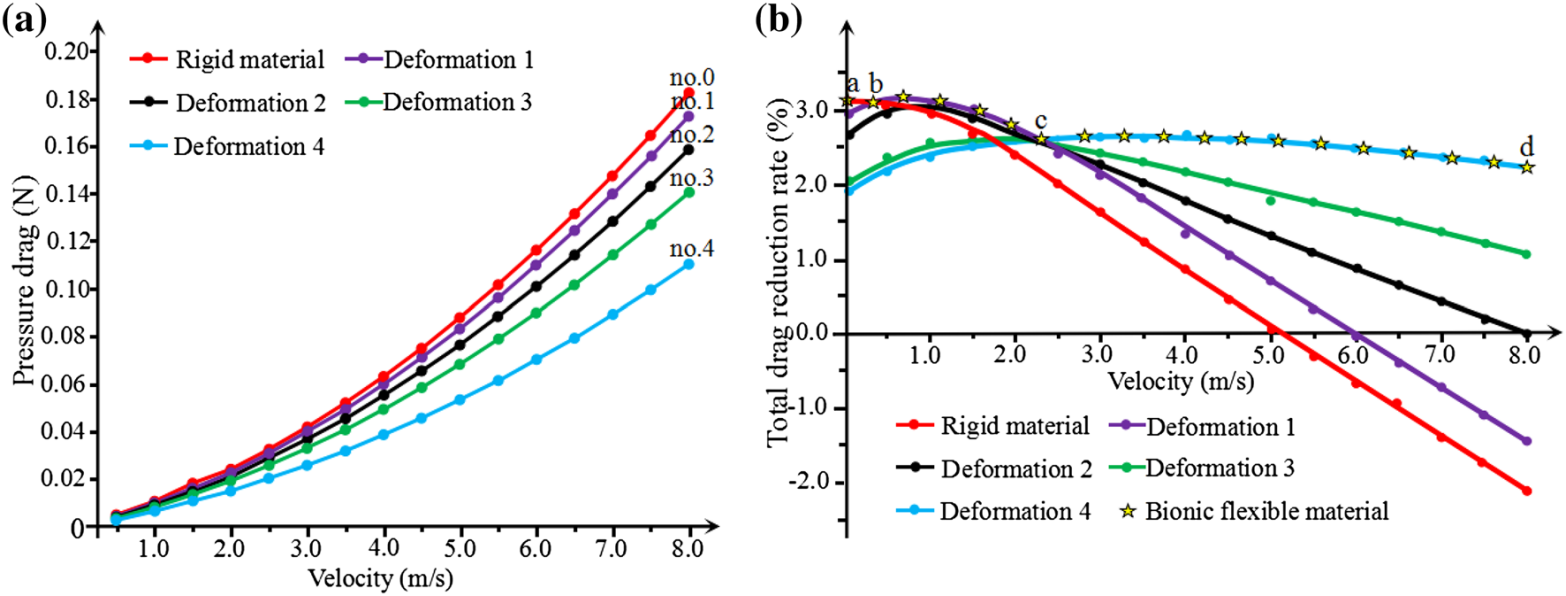
Analysis of flexible deformation. (**a**) Pressure drag; (**b**) total drag reduction rate under different deformations.

Figure 6 shows the different flexible deformations of bionic grooves. The pressure drag of surface is numerically analyzed. From the results shown in Fig. 7a , the pressure drags also increases gradually with flow velocity under all deformations. However, among the deformations, the pressure drag of the largest flexible deformation (D4) is the lowest. Eventually, the flexible surface helps reduce the pressure drags through its self-adaptive character.

Figure 7b shows drag reduction rate of the flexible surface under different deformations. The variation tendency indicates that the values increases at the beginning stage, and then decreases until to a minus drag reduction rate; namely, the drag increasing effect would disappear unless greater deformation happened. For example, the rigid material surface will lost the drag reducing function at 5 m/s velocity point, and increase drag after 5 m/s. Therefore, the material need a real-time deformation to suit to the flow velocity and make the fluid pass easily. The deformation D4 that shows relative higher level of drag reduction rate and wider flow velocity range.

For a self-adaptive trait of flexible material, when a hypothetical flow passing on this flexible surface, a dynamical variation will happen and real-time drag reduction will combine of subsections ab, bc and cd (Fig. 7b). The average value will definitely be maximized. Meanwhile, the velocity range of drag reduction was also expanded by the flexible material.

## Conclusions

In this paper, the distribution microscopic morphology and structure characteristics of loach scales are observed and analyzed by using 3D microscope and SEM. It has been found that the adjacent scales on loach body surface are arranged in a fan-shaped overlapping style. Each scale is composed of four regions, namely apical, focal, basal and lateral. Each zone is covered with micro groove structures, which are embedded in the skin tissue in the focal and basal regions. The marvellous groove-like strips covers on the apical part, and such area directly contacts with the incoming flow and generates frictional resistance. The structure of groove-like stripes within the apical zone is affected by the remaining mucus, which can avoid or slow down the loss of mucus and reduce the swimming resistance.

The drag reduction mechanism is revealed through our numerical analysis. The pressure near the wall of bionic surface is found lower than the surrounding, which created a “water absorption” area and formed a stable lubricating water film near the wall surface. The thickness of water membrane on the bionic surface was more thicker than that on the smooth surface, and consequently the velocity gradient, turbulence intensity and turbulence kinetic energy were reduced. Its shear resistance was less than that of the smooth surface. On the other hand, a number of clockwise vortex were generated in micro architectures, acting as anti-friction bearings.

In addition, the flexible body had marvelous self adaptive characteristic which could reduce the pressure drag through real time deformation, and kept the drag reduction rate at relative level in more wider velocity range.
